# The Role of Reactive Oxygen Species and Autophagy in Periodontitis and Their Potential Linkage

**DOI:** 10.3389/fphys.2017.00439

**Published:** 2017-06-23

**Authors:** Chengcheng Liu, Longyi Mo, Yulong Niu, Xin Li, Xuedong Zhou, Xin Xu

**Affiliations:** ^1^State Key Laboratory of Oral Disease, West China Hospital of Stomatology, Sichuan UniversityChengdu, China; ^2^Department of Periodontics, West China Hospital of Stomatology, Sichuan UniversityChengdu, China; ^3^Key Lab of Bio-resources and Eco-environment of Ministry of Education, College of Life Sciences, Sichuan UniversityChengdu, China; ^4^Institute of Biophysics, Chinese Academy of SciencesBeijing, China; ^5^Department of Operative Dentistry and Endodontics, West China Hospital of Stomatology, Sichuan UniversityChengdu, China

**Keywords:** periodontitis, reactive oxygen species, autophagy, NF-κB, JNK, mTORC1, Beclin 1, Atg12-Atg5 complex

## Abstract

Periodontitis is a chronic inflammatory disease that causes damage to periodontal tissues, which include the gingiva, periodontal ligament, and alveolar bone. The major cause of periodontal tissue destruction is an inappropriate host response to microorganisms and their products. Specifically, a homeostatic imbalance between reactive oxygen species (ROS) and antioxidant defense systems has been implicated in the pathogenesis of periodontitis. Elevated levels of ROS acting as intracellular signal transducers result in autophagy, which plays a dual role in periodontitis by promoting cell death or blocking apoptosis in infected cells. Autophagy can also regulate ROS generation and scavenging. Investigations are ongoing to elucidate the crosstalk mechanisms between ROS and autophagy. Here, we review the physiological and pathological roles of ROS and autophagy in periodontal tissues. The redox-sensitive pathways related to autophagy, such as mTORC1, Beclin 1, and the Atg12-Atg5 complex, are explored in depth to provide a comprehensive overview of the crosstalk between ROS and autophagy. Based on the current evidence, we suggest that a potential linkage between ROS and autophagy is involved in the pathogenesis of periodontitis.

## Introduction

Periodontitis is an inflammatory disease that compromises the integrity of the tooth-supporting tissues through the interplay of periodontal pathogens and the host immune response (Kinane et al., 2008; Dumitrescu, 2016). A new model of the pathogenesis of periodontitis showed that pathogens alone are necessary but insufficient for the development of periodontal lesions *per se*. The majority of periodontal tissue damage is caused by the subversion of host immune responses, with the involvement of leukocytes, complement and reactive oxygen species (ROS) (Hajishengallis, 2015). ROS are short-lived, highly reactive reduced products of oxygen, such as superoxide (${\text{O}}_{2}^{-}\cdot$), hydrogen peroxide (H_2_O_2_), the hydroxyl radical (·OH), and singlet oxygen (^1^O_2_) (Di Meo et al., 2016). The close relationship between ROS and periodontitis has long been appreciated, beginning with the pioneering studies of the early 1970s (Shapira et al., 1991; Marquis, 1995; Chapple, 1997). The role of ROS in periodontitis has been comprehensively reviewed (Chapple and Matthews, 2007; Nibali and Donos, 2013). In brief, ROS at the cellular level are essential for physiologic processes of eukaryotic cells, including cellular signaling transduction, cellular differentiation, and apoptosis (McClean et al., 2015; Di Meo et al., 2016). Moreover, ROS contribute to the oxidative killing of pathogens (Roos et al., 2003). For instance, a clinical study found that levels of serum reactive oxygen metabolites were positively correlated with immunoglobulin G antibodies to specific periodontal pathogens, including *Porphyromonas gingivalis* (*P. gingivalis*), *Aggregatibacter actinomycetemcomitans* (*A. actinomycetemcomitans*), and *Prevotella intermedia* (*P. intermedia*) (Tamaki et al., 2014). However, a homeostatic imbalance between ROS and antioxidant defense systems can trigger an oxidative stress response, which is believed to be related to periodontal destruction (Waddington et al., 2000; Baltacioglu et al., 2014b). Clinically, there are strong positive correlations between periodontal parameters and malondialdehyde (MDA) and total oxidant status (TOS) levels (Akalin et al., 2007; Baltacioglu et al., 2014b). Further evidence has been derived from *in vitro* and animal model studies. Decreased ROS levels downregulated the expression of osteoclast differentiation marker genes and attenuated bone loss (Kanzaki et al., 2013). ROS can also evoke immune responses through redox-sensitive gene transcription factors such as nuclear factor kappa-light-chain-enhancer of activated B cells (NF-κB) (Gan et al., 2016). In addition, ROS can induce cellular apoptosis via c-Jun N-terminal kinase (JNK) activation (Liu et al., 2015).

Accumulating evidence has indicated a close connection between ROS and autophagy. A series of discoveries related to autophagy won Yoshinori Ohsumi the Nobel Prize for Physiology and Medicine in 2016 (Levine and Klionsky, 2016; Tooze and Dikic, 2016). Autophagy is a lysosomal degradation pathway of self-digestion (Klionsky and Emr, 2000; Yang and Klionsky, 2010; Levine and Klionsky, 2016). This process is thought to have evolved as a stress response that allows organisms to survive harsh conditions (Mizushima et al., 1998; Netea-Maier et al., 2016). There is a complex, reciprocal relationship between the autophagy pathway and ROS. Studies suggest that ROS influence autophagy and that autophagy reciprocally regulates ROS (He Y. et al., 2017; Wang et al., 2017). The most typical example of their interaction has been elucidated in cancer development (Zhao et al., 2016). Autophagy consists of five sequential steps: induction, elongation, maturation, transport to lysosomes, and degradation (Levine and Kroemer, 2008). Thus, the function of autophagy is step dependent. The regulation of autophagy by ROS appears to be complicated, involving various autophagic signaling pathways and autophagy-related genes (Atgs). Studies have clearly demonstrated that the regulation of autophagy by ROS plays both a cytoprotective and cytotoxic role in cancer development (Chen et al., 2016; Zhong et al., 2016). Recently, autophagy has been proposed to be involved in the pathogenesis of periodontitis through bacterial elimination, facilitating the internalization of specific periodontal pathogens, suppressing the immune response, and inhibiting periodontal cell apoptosis (Tsuda et al., 2010; An et al., 2016; Tan et al., 2016; Park et al., 2017).

ROS and autophagy are closely interconnected, and many key molecules are shared by the two processes. However, the available data suggest that the intricate interactions between ROS and autophagy in periodontitis remain unknown. Moreover, the mechanisms underlying how ROS participate in regulating autophagy remain to be elucidated. To contribute to the understanding of this issue, the present review focuses on redox-sensitive pathways and transcription factors related to autophagy and summarizes the physiologic and pathologic roles of oxidative stress and autophagy in periodontal tissues.

## ROS homeostasis

In general, ROS include ${\text{O}}_{2}^{-}\cdot$, H_2_O_2_, ·OH, and ^1^O_2_ (Di Meo et al., 2016). These species are endogenously generated by peroxisomes; the endoplasmic reticulum (ER); enzymes such as NADPH oxidases (NOXs), xanthine oxidases (XOs), cyclooxygenases (COXs) and lipoxygenases (LOXs); and the mitochondrial electron transport chain (Mito-ETC) (Zhang L. et al., 2015). The amount of intracellular ROS produced daily reaches ~1 billion molecules in every single cell. It is commonly accepted that the Mito-ETC is the major source of ROS (Filomeni et al., 2015). In the ETC, electrons are transferred from electron donors (e.g., NADH) to electron acceptors (e.g., O_2_) via redox reactions, resulting in the synthesis of adenosine triphosphate (ATP). In mitochondria, premature electrons leakage to O_2_ can occur, generating ${\text{O}}_{2}^{-}\cdot$ as a by-product of the ETC. Dismutation of ${\text{O}}_{2}^{-}\cdot$ by superoxide dismutase 1 (SOD1, also known as CuZn-SOD) in the intermembrane space, or by superoxide dismutase 2 (SOD2, also known as MnSOD) in the matrix, produces H_2_O_2_. In turn, H_2_O_2_ is reduced to H_2_O by glutathione peroxidase (GPX) or catalase (CAT) (Perrone et al., 2016). H_2_O_2_ is partially degraded to ·OH in the Fenton and Haber-Weiss reactions (Turrens, 2003). The components of the ETC are organized into four complexes. ROS generation by the ETC is primarily dependent on complex I (also known as NADH-coenzyme Q reductase or NADH dehydrogenase) and complex III (also known as coenzyme Q reductase) (Lismont et al., 2015).

The generation of ROS occurs in equilibrium with a wealth of ROS scavengers, including enzymes (e.g., SOD, GPX, and CAT), small molecules [e.g., vitamin C and glutathione (GSH)], and glutaredoxin and thioredoxin systems, to maintain redox homeostasis (Venditti et al., 2013; Netto and Antunes, 2016). The balance between the generation and elimination of ROS is critical for human health. Excessive production of ROS, low levels of antioxidants, or inhibition of antioxidant enzymes causes oxidative stress and may lead to indiscriminate damage to biological macromolecules (lipids, proteins, and DNA). Increasing evidence has shown an association between ROS and a variety of diseases, including cancer, periodontitis, cardiovascular diseases, and diabetes (Di Meo et al., 2016).

## ROS in periodontitis

ROS are considered to be a double-edged sword in periodontal diseases (Nibali and Donos, 2013). At low concentrations, ROS stimulate the proliferation and differentiation of human periodontal ligament fibroblasts (hPDLFs) in culture, while at higher concentrations, they may have cytotoxic effects on periodontal tissues and participate in pathogen killing (Chapple and Matthews, 2007; D'aiuto et al., 2010; Galli et al., 2011; Saita et al., 2016).

### The physiologic role of ROS in periodontal tissues

Periodontitis is a disease caused by oral infection associated with polymicrobial dysbiosis and the activation of host immunity (Hajishengallis, 2014). Keystone or keystone-like pathogens, such as *P. gingivalis* and *Tannerella forsythia* (*T. forsythia*), can drive the disruption of periodontal tissue homeostasis and lead to inflammation (Wright et al., 2014; Lamont and Hajishengallis, 2015). Keystone or keystone-like pathogens of periodontitis, predominantly Gram-negative anaerobic or facultative bacteria, are appreciably sensitive to changes in the oxidative environment (Lamont and Hajishengallis, 2015). ROS can disturb the cellular oxidative environment and participate in the killing of keystone pathogens. For instance, a marked increase in ROS generation was observed when leukocytes were treated with *P. gingivalis* lipopolysaccharide (LPS) or *Fusobacterium nucleatum* (*F. nucleatum*) *in vitro* (Sheikhi et al., 2000; Zhu et al., 2016). Fascinatingly, it has very recently been reported that H_2_O_2_ is a central determinant of oral polymicrobial synergy (Lamont, 2016). However, several lines of evidence have suggested that periodontal pathogens such as *Treponema denticola* (*T. denticola*) have evolved strategies to suppress the induction of ROS (Shin et al., 2013).

Conversely, at basal levels, ROS serve as second messenger particulates in regulating signal transduction, cellular homeostasis, and cell death. For instance, H_2_O_2_ can trigger defensive inflammatory responses to environmental cues in periodontal tissues through mitogen-activated protein kinase (MAPK) and NF-κB as well as inflammasome activation (Almerich-Silla et al., 2015). Moreover, glucose oxidase, which continuously generates H_2_O_2_ at relatively low concentrations, could stimulate the proliferation and osteoblastic differentiation of hPDLFs through the upregulation of runt-related transcription factor-2 (Runx2) and osterix (Choe et al., 2012). H_2_O_2_could also increase the levels of gelatinolytic matrix metalloproteinases (MMPs), enhancing hPDLF migration in an MMP-dependent manner (Cavalla et al., 2015). These findings suggest that ROS participate in the proliferation and differentiation of hPDLFs. However, many studies have reported that H_2_O_2_ acts predominantly as an inhibitory mediator of cell proliferation and differentiation (Choi et al., 2009). A possible explanation for these contradictory results is that cellular responses to H_2_O_2_ can differ depending on the concentration of H_2_O_2_ and the type of cells. For example, Burdon et al. reported that exposure to 1 μM H_2_O_2_ promoted the proliferation of BHK-21 fibroblasts, while H_2_O_2_ at 0.5 and 1 mM caused apoptotic cell death (Burdon et al., 1996).

### ROS in periodontal pathogenesis

ROS have multifaceted effects, and the function of ROS is determined by the redox state (Zhao et al., 2016). Oxidative stress is induced when ROS are produced in excess of the capacity of the antioxidant system to efficiently counteract their actions, resulting in cytotoxic effects and enhancing periodontal destruction (Nibali and Donos, 2013). The involvement of ROS in the pathogenesis of periodontal diseases is highlighted by the existence of a disturbed redox balance in periodontitis cases. The results of recently published relevant studies have been summarized in Table 1. Data from a few cross-sectional studies have demonstrated low plasma and serum total antioxidant (TAOC) concentrations in periodontitis patients relative to healthy controls (Chapple et al., 2002; Brock et al., 2004; D'aiuto et al., 2010; Baltacioglu et al., 2014a; Thomas et al., 2014; Baser et al., 2015; Patil et al., 2016). Saliva is well recognized as containing a pool of markers for periodontitis (Zhang et al., 2016). Studies have also found similar results regarding salivary TAOC. The salivary TAOC was significantly lower in patients with chronic periodontitis compared with healthy controls (Diab-Ladki et al., 2003; Baltacioglu et al., 2014a; Miricescu et al., 2014; Baser et al., 2015; Zhang T. et al., 2015). Moreover, higher levels of reactive oxygen metabolites and total oxidant status (TOS) were observed in the serum, saliva, and gingival crevicular fluid (GCF) of patients with periodontitis compared with controls (Akalin et al., 2007; D'aiuto et al., 2010; Wei et al., 2010; Baltacioglu et al., 2014a,b). Furthermore, there is a strong negative correlation between salivary TAOC and clinical attachment loss (CAL) in periodontitis patients (Baser et al., 2015). Significant positive correlations were also observed between malondialdehyde (MDA), an LPO product, and TOS levels and periodontal parameters (Akalin et al., 2007). Collectively, these results suggested that reduced TAOC and increased ROS may be risk factors for periodontitis or may be caused by periodontal inflammation. However, it is very difficult to determine whether the change in redox status is the cause or a result of periodontitis. In addition, as summarized in Table 1, decreased levels of specific antioxidants, such as SOD, CAT, and GPX, were observed in periodontitis patients compared with healthy controls (Panjamurthy et al., 2005; Wei et al., 2010; Trivedi et al., 2014; Patil et al., 2016).

**Table 1:** Levels of oxidative stress and antioxidant parameters in periodontitis patients compared with healthy ones.

Reactive oxygen species	Total oxidant levels	Diacron reactive oxygen metabolites (D-ROM)	Increase in serum (D'aiuto et al., 2010)
		Total oxidant status (TOS)	Increase in serum, saliva, and GCF (Akalin et al., 2007; Wei et al., 2010; Baltacioglu et al., 2014a,b)
Antioxidants	Total antioxidant levels	3-ethylbenzothiazoline 6-sulfonate (ABTS) reduction assays	Decrease in saliva (Diab-Ladki et al., 2003; Miricescu et al., 2014; Zhang T. et al., 2015)
		Plasma biological antioxidant potential (BAP) assay	Decrease in serum (D'aiuto et al., 2010)
		Ferric reducing antioxidant power (FRAP) assay	Decrease in serum and saliva (Baltacioglu et al., 2014b)
		Enhanced chemiluminescent (ECL) assay	Decrease in plasma (Chapple et al., 2002; Brock et al., 2004) and GCF (Chapple et al., 2002)
		Total blood antioxidant capacity (NBT test)	Decrease in serum (Thomas et al., 2014)
	Specific antioxidants	Superoxide dismutase (SOD)	Decrease in RBC lysate and saliva (Trivedi et al., 2014; Patil et al., 2016) Increase in serum, saliva, and GCF (Wei et al., 2010) Increase in plasma and tissue (Panjamurthy et al., 2005)
		Catalase (CAT) activity	Decrease in RBC lysate and saliva (Trivedi et al., 2014; Patil et al., 2016) Increase in plasma and tissue (Panjamurthy et al., 2005)
		Reduced and oxidized glutathione (GSH and GSSG)	Decrease in saliva (Tsai et al., 2005), GCF (Chapple et al., 2002), blood (Panjamurthy et al., 2005), and tissue (Panjamurthy et al., 2005)
		Glutathione peroxidase (GPX)	Decrease in RBC lysate (Trivedi et al., 2014) and saliva (Miricescu et al., 2014; Trivedi et al., 2014) No significant change in saliva (Tsai et al., 2005)
		Vitamin C	Decrease in plasma (Panjamurthy et al., 2005; Patil et al., 2016) and tissue (Panjamurthy et al., 2005)
		Vitamin E	Decrease in plasma and tissue (Panjamurthy et al., 2005)

Comprehensive reviews on tissue damage caused by ROS have been published (Waddington et al., 2000; Chapple and Matthews, 2007; Nibali and Donos, 2013). To summarize, periodontal tissue damage may arise directly from oxidative stress and indirectly via the activation of cell signaling pathways related to inflammation, apoptosis, and other factors. It has been demonstrated that direct tissue damage caused by ROS can be mediated by (1) the induction of lipid peroxidation and cell membrane destruction (Mashayekhi et al., 2005; Panjamurthy et al., 2005; Tsai et al., 2005; Pradeep et al., 2013), which results in (2) protein denaturation and enzyme deactivation (Nibali and Donos, 2013; Trivedi et al., 2014; Nguyen et al., 2016; Patil et al., 2016), leading to (3) nucleic acid damage (e.g., strand breaks and base pair mutations) and chromosome disruption (Takane et al., 2002) and causing (4) mitochondrial injury and ROS bursts (Battino et al., 1999). Tissue destruction can be assessed by measuring the levels of markers for lipid peroxidation, protein damage, and DNA damage, such as MDA, protein carbonylation and 8-hydroxy-2-deoxyguanosine (8-OHdG) (Sawamoto et al., 2005; Takane et al., 2005; Canakci et al., 2009; Su et al., 2009; Mai et al., 2012; Sezer et al., 2012; Dede et al., 2013; Hendek et al., 2015). The results of relevant published studies have been summarized in Table 2.

**Table 2:** Levels of markers of oxidative stress damage in periodontitis patients compared with healthy ones.

**Types**	**Markers**	**Expression levels**
Lipid damage	Lipid peroxidation (TBARS assay)	Increase in saliva (Mashayekhi et al., 2005; Tsai et al., 2005), plasma (Panjamurthy et al., 2005) and tissue (Panjamurthy et al., 2005)
Protein damage	Malondialdehyde (MDA)	Increase in plasma (Trivedi et al., 2014), RBC lysate (Patil et al., 2016), saliva (Akalin et al., 2007; Khalili and Biloklytska, 2008; Baltacioglu et al., 2014a; Miricescu et al., 2014; Trivedi et al., 2014; Nguyen et al., 2016), and GCF (Akalin et al., 2007; Wei et al., 2010) No significant change in serum (Akalin et al., 2007; Baltacioglu et al., 2014b)
	Protein carbonylation	Increase in saliva (Su et al., 2009; Nguyen et al., 2016)
DNA damage	8-hydroxy-2-deoxyguanosine (8-OHdG)	Increase in saliva (Takane et al., 2002, 2005; Sawamoto et al., 2005; Canakci et al., 2009; Su et al., 2009; Sezer et al., 2012; Miricescu et al., 2014; Nguyen et al., 2016) and GCF (Hendek et al., 2015) No significant change in saliva (Dede et al., 2013)
	Leukocyte telomere lengths (LTL)	LTL was negatively correlated with oxidative stress (*P* = 0.008); and severity of periodontitis (*P* = 0.003; *R* = −0.2) (Masi et al., 2011)

**Figure 1:**
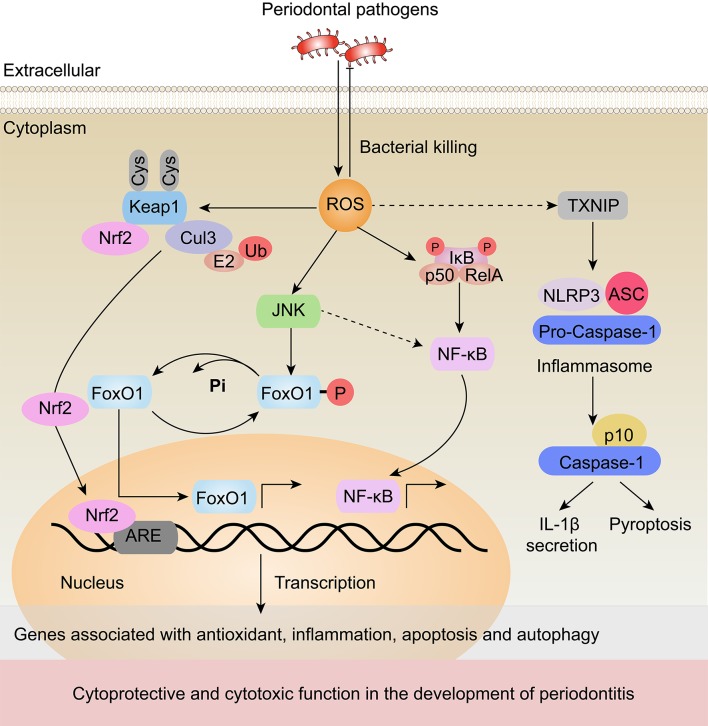
Underlying signaling pathways of ROS regulation in periodontitis. Periodontal pathogen infection can promote ROS generation. In turn, ROS can contribute to the oxidative killing of the pathogens. ROS generated from mitochondria activate the transcription of genes associated with inflammation, apoptosis and autophagy through JNK, NF-κB, and inflammasome-dependent signaling pathways, which leads to cytoprotective and cytotoxic effects in the development of periodontitis. (1) ROS activate JNK, which results in the dephosphorylation of FoxO1. (2) ROS have been shown to activate NF-κB in periodontitis. (3) ROS promote excessive inflammation by activating TXNIP, which subsequently activates the NLRP3 inflammasome, elevates the secretion of its substrates, such as IL-1β, and induces pyroptosis. (4) Meanwhile, ROS interact with cysteine residues in Keap1, disrupting the Keap1-Cul3 ubiquitination system and leading to the release of Nrf2 to the nucleus. In the nucleus, Nrf2 binds to AREs to initiate the transcription of a number of antioxidant genes. Black arrows (↑) and perpendicular lines (⊥) denote activation and suppression, respectively. Dashed lines denote regulatory relationships that need to be confirmed in periodontitis.

A more complex question is how ROS result in periodontal tissue damage by regulating signal transduction and gene transcription, which is described in Figure 1. There are at least four pathways relevant to this topic. First, ROS are able to activate NF-κB, initiating a signaling cascade that regulates inflammatory and immune responses (Morgan and Liu, 2011). Second, ROS are involved in inducing JNK activation, resulting in cell apoptosis (Nakano et al., 2006). Third, ROS are associated with inflammasome activation, leading to pyroptic cell death (Zhou et al., 2010). Fourth, ROS play a critical role in autophagy (Filomeni et al., 2015). This section will focus on evidence for the mechanisms of ROS-mediated activation of NF-κB, JNK, and inflammasomes in periodontitis. The relationship between ROS and autophagy in periodontitis will be described later.

ROS have been reported to both activate and repress NF-κB signaling in studies of different cells and different upstream pathways, and ROS-mediated activation of NF-κB signaling results in the expression of pro-inflammatory cytokines and chemokines (Ozcan et al., 2016). The expression of these cytokines leads to periodontal destruction by triggering inflammatory responses and osteoclastic differentiation (Hans and Hans, 2011; Souza and Lerner, 2013). This effect can be inhibited by exogenously added antioxidants. For example, when intracellular ROS were scavenged during receptor activator for nuclear factor-κB ligand (RANKL)-stimulated osteoclastogenesis, the RANKL-induced activation of NF-κB was abrogated (Nikhil et al., 2015). Furthermore, Thummuri et al. demonstrated that thymoquinone, an antioxidant, could inhibit inflammation-induced ROS generation and the activation of NF-κB in osteoclast precursors (Thummuri et al., 2015).

ROS can also trigger JNK signaling during periodontitis (Wang et al., 2015; Lee et al., 2016). A recent study showed that ROS induced the activation of JNK signaling, which disrupted the periodontal junctional epithelium through the dissociation of E-cadherin (Wang et al., 2015; Lee et al., 2016). Consistent with this result, nicotine-induced ROS generation induced JNK phosphorylation in human gingival fibroblasts (HGFs). Furthermore, constitutive activation of JNK initiated the apoptosis cascade via the caspase-3-dependent pathway (Kang et al., 2011). In contrast to the pro-apoptotic function of JNK in HGFs, another study demonstrated an anti-apoptotic role of JNK in response to bacterial invasion (Wang et al., 2015). This study reported that JNK activation could induce the expression of genes that counter oxidative stress (Cat, Sod2, Prdx3) and apoptosis (Bcl-6) via the activation of the transcription factor forkhead box protein O1 (FoxO1) (Wang et al., 2015). Collectively, these results suggest that the activation of JNK in periodontal cells mediates cell survival, and this function may be condition and cell type dependent.

Another mechanism by which ROS are involved in periodontal pathogenesis is via the activation of inflammasomes. As previously reported, ROS induced the activation of NLRP3 by causing thioredoxin (TRX)-interacting protein (TXNIP) to dissociate from thioredoxin, which may be associated with periodontitis (Schroder et al., 2010; Zhou et al., 2010). Increasing clinical data support this point. Bostanci et al. first reported significantly high levels of NLRP3 as well as enhanced release of pro-inflammatory cytokines (IL-1β and IL-18) in patients with periodontitis compared with healthy controls (Bostanci et al., 2009). Elevated IL-1β and IL-18 levels could contribute to the triggering of periodontal destruction. Consistent with this study, Xue et al. and Huang et al. demonstrated increased levels of NLRP3 in the gingival tissues of periodontitis patients compared with healthy individuals via real-time PCR and immunohistochemistry (Huang et al., 2015; Xue et al., 2015). The activation of NLRP3 inflammasomes via ROS can lead to IL-1β secretion and pyroptosis.

Emerging evidence has indicated that nuclear factor erythroid 2-related factor 2 (Nrf2) plays an important cytoprotective role in oxidative-stress-associated periodontal damage. As a redox-sensitive factor, Nrf2 protects cells against cytotoxic ROS. As illustrated in Figure 1, oxidative stress disrupts critical cysteine residues in Kelch like-ECH-associated protein 1 (Keap1). When Nrf2 is not ubiquitinated, it dissociates from Keap1, translocates into the nucleus and binds to antioxidant response elements (AREs) to initiate the transcription of antioxidant genes, such as heme oxygenase-1 (HO-1), SOD, and CAT (Ma, 2013). Nrf2 knockout exacerbated the loss of periodontal tissues in a mouse model of periodontitis. In addition, an analysis of oral polymorphonuclear neutrophils (oPMNs) and blood PMNs revealed that Nrf2 expression was significantly decreased in patients with severe chronic periodontitis compared with periodontally healthy controls (Sima et al., 2016). Moreover, compared with blood PMNs, thirty Nrf2 pathway-related genes were differentially expressed in oPMNs from chronic periodontitis patients (Sima et al., 2016). These results indicated that Nrf2 and its downstream genes may be involved in the pathological process of periodontitis via their antioxidative effects.

Sirtuins (silent information regulator, Sir2) belong to a conserved family of nicotinamide adenine dinucleotide (NAD)-dependent protein deacylases. There are seven human Sir2 homologs, SIRT1 through SIRT7 (Chalkiadaki and Guarente, 2015). Increasing evidence has indicated that SIRT activation suppresses oxidative stress (Kumar et al., 2017), and the expression of SIRT1 was shown to be increased at the gene and protein levels in LPS-treated human periodontal ligament cells (Park et al., 2012). However, the direct linkage between SIRT and oxidative stress in periodontitis has not been elucidated.

## Autophagy activation in periodontitis

Autophagy is an evolutionarily conserved intracellular degradation system that delivers damaged or superfluous cytoplasmic material (e.g., damaged organelles, denatured proteins, and bacteria) to the lysosome and recycles degradation products for new synthesis or energy production (Filomeni et al., 2015). In a broad sense, there are four different forms of autophagy: macroautophagy, microautophagy, chaperone-mediated autophagy (CMA) and non-canonical autophagy. Among these forms, macroautophagy (hereafter referred to as autophagy) is the most widely investigated type (Kabat et al., 2016). Unlike other intracellular degradation pathways, autophagy sequesters intracellular material inside a double-membrane vesicle called the autophagosome. Subsequently, the autophagosome fuses with lysosomes, resulting in the degradation of the vesicle (Shibutani et al., 2015). The complete autophagy process can be divided into five highly regulated stages, including induction, elongation, maturation, transport to lysosomes, and degradation (Tooze and Dikic, 2016). Periodontitis is a multifactorial inflammatory disease (Dumitrescu, 2015). Periodontal pathogens residing in dental plaques and the periodontal pocket are believed to be the primary etiology of periodontitis (Hajishengallis, 2015). In previous studies, depending on context, the induction of autophagy has been shown to have both protective and pathological effect in periodontitis. *Song* et al. have comprehensively reviewed the role of autophagy in periodontitis (Song et al., 2016). In summary, autophagy may participate in periodontitis via the following mechanisms: (1) regulating periodontal pathogen invasion; (2) regulating immune signaling, resulting in inflammatory disorders and periodontal tissue damage; and (3) protecting periodontal cells from apoptosis.

### Autophagy in periodontal pathogen invasion

Abundant evidence has demonstrated that periodontitis is highly associated with microbial infection. As an intracellular innate immune defense pathway, autophagy is usually enhanced in infected cells, contributing to antimicrobial defense mechanisms. Autophagy can eliminate intracellular pathogens such as *Mycobacterium tuberculosis* (*M. tuberculosis*) (Kim et al., 2012). To avoid lysosomal killing, many pathogens, including *Legionella pneumophila* (*L. pneumophila*), have developed strategies to suppress cellular autophagy. However, findings obtained via *in vitro* experiments in which cultured cells were exposed to bacteria have suggested that periodontal pathogens such as *P. gingivalis* participate in the induction of autophagy. Belanger et al. found that *P. gingivalis* trafficked quickly from phagosomes to autophagosomes in human coronary artery endothelial cells (Belanger et al., 2006). This result is consistent with the finding that ROS generated by *P. gingivalis* contribute to increased levels of LC3 proteins and promoting the conversion of LC3-I to LC3-II (Park et al., 2017). Taken together, these results strongly suggest that the induction of autophagy can facilitate specific periodontal bacterial survival by replication within an autophagosome-like compartment. However, no LC3 lipidation was found when cells were infected with *A. actinomycetemcomitans* (Blasi et al., 2016), suggesting that the activity of cellular autophagy in response to infection is associated with periodontal bacterial species.

### Autophagy in the periodontal immune response and inflammation

The relationship between autophagy and immunity has been systematically reviewed in the published literature (Levine et al., 2011; Deretic et al., 2013; Shibutani et al., 2015). Here, we mainly focus on the potential immune consequences of autophagy for periodontitis. Autophagy functions as a modulator of classical pattern recognition receptors (PRRs), such as Toll-like receptors (TLRs), and Nod-like receptors (NLRs), regulating the periodontal innate immune response (Deretic et al., 2013; Oh and Lee, 2014). Furthermore, autophagy can suppress the periodontal immune response by inhibiting cytokine secretion. First, autophagy plays a negative role in inflammasome activation and secretion of IL-1β and IL-18. Animal studies have shown that mice lacking LC3B produced higher levels of caspase-1-dependent cytokines than wild-type mice. Similar results were found in LC3B-deficient macrophages (Nakahira et al., 2011). LC3B is a ubiquitin-like protein that participates in autophagosome formation and maturation (Anton et al., 2016). Second, autophagy negatively regulates the secretion of IL-1α. Castillo et al. found that mice lacking Atg5 produced more IL-1α via a ROS-calpain pro-inflammatory pathway (Castillo et al., 2012). It therefore seems reasonable to consider that autophagy might influence periodontal inflammation by regulating both inflammasome-dependent and inflammasome-independent inflammation.

### Autophagy protects periodontal cells from apoptosis

Studies have shown that the inhibition of autophagy in HGFs treated with *P. gingivalis* LPS induced apoptosis, suggesting a protective role of autophagy (Bullon et al., 2012). To further explore the role of autophagy in periodontitis, a recent study measured the expression of LC3 and observed autophagic vacuoles in periodontal ligament (PDL) tissues from individuals with and without periodontitis. The results showed increased LC3 expression and autophagosome production in inflammatory PDL tissues (An et al., 2016). In addition, co-localization of LC3 and melanoregulin (MREG) was found in gingival epithelial cells isolated from severe periodontal disease-affected individuals, while this effect was absent in cells from healthy or moderately affected individuals (Blasi et al., 2016). As multiple studies have proposed that autophagy may antagonize apoptosis, these results suggest a potential protective role of autophagy in periodontal tissues. However, whether the blocking of autophagy induces apoptosis in periodontal tissues remains unknown.

## Redox regulation of autophagy in periodontitis

**Figure 2:**
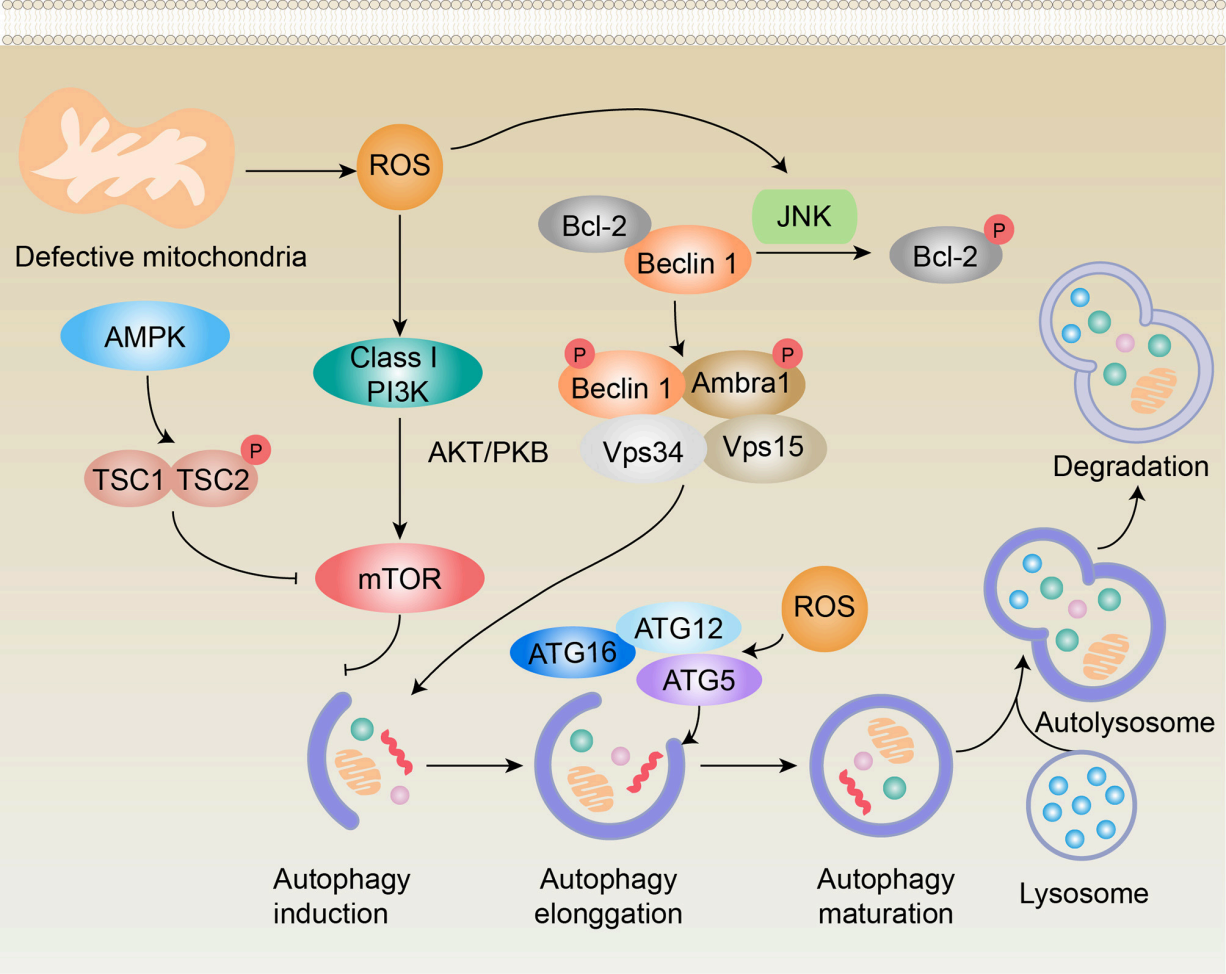
Schematic representation of potential pathways of redox regulation of autophagy in periodontitis. ROS regulate autophagy via at least four different mechanisms, including (1) the phosphorylation of Bcl-2 by JNK in a ROS-dependent manner that leads to Beclin 1 dissociation and autophagy induction; (2) initiation of the PI3K-AKT pathway, resulting in the activation of mTOR, which functions as an inhibitor of autophagy induction; (3) inhibition of TORC1 activity in an AMPK-dependent manner, contributing to the activation of autophagy; and (4) activation of the Atg12-Atg5 complex, which promotes autophagy elongation. Black arrows (↑) and perpendicular lines (⊥) denote activation and suppression, respectively.

Mitochondrial ROS have been identified as important signaling molecules in regulating autophagy. Moreover, bacterial infection induces the generation of ROS (Golz et al., 2014). Elevated ROS can regulate autophagy activity by targeting autophagy-related genes (Atgs) and/or upstream signaling pathways, including mammalian target of rapamycin complex 1 (mTORC1), Beclin 1, and the Atg12-Atg5 complex, as outlined in Figure 2. Emerging evidence has suggested the involvement of ROS-autophagy reciprocity in periodontitis. The expression of autophagy-related genes (Atg12 and LC3) was shown to be positively correlated with mitochondrial ROS production in peripheral blood mononuclear cells from patients with periodontitis (Bullon et al., 2012). Furthermore, a reduction of mitochondrial ROS induced a decrease in autophagy (Bullon et al., 2012).

### ROS disrupts autophagy induction by interfering with mTORC1

The activity of mTORC1 is regulated by numerous input signals, such as rapamycin, insulin, and oxidative stress. Studies have found that ROS could influence mTORC1 activity through the tuberous sclerosis complex 1/2 (TSC1/TSC2) heterodimer. Increased levels of ROS activate AMP-activated protein kinase (AMPK), which causes TSC2 phosphorylation and activates the TSC1/TSC2 complex, thus inhibiting mTORC1 and stimulating ULK (an important initiator of the autophagy complex) to induce autophagy (Yu et al., 2010; Zhang et al., 2013; Zhang J. et al., 2015). Conversely, ROS can activate the phosphoinositide-3-kinase (PI3K)-protein kinase B (Akt)-mTORC1 signaling pathway by directly activating PI3K or by regulating the phosphorylation state of Akt, thus inhibiting autophagy induction (Dermit et al., 2016; Su et al., 2017). Stafford *et al*. found that invasion of *P. gingivalis* inhibited the mTOR pathway in oral epithelial cells, which was the first reported evidence to suggest a potential role for mTORC1 in periodontitis (Stafford et al., 2013).

### ROS inhibits autophagic flux by targeting beclin 1

As noted above, increased levels of ROS can activate NF-κB, which may result in the upregulation of Atgs, including Beclin 1 (He Z. J. et al., 2017). Moreover, the activation of JNK signaling during oxidative stress leads to the phosphorylation of Bcl-2, which causes Beclin 1 to dissociate from the Vps34 complex and results in the activation of autophagy (Ni et al., 2014). Several studies have shown the relevance of these Atgs in periodontitis. Specifically, there were higher protein expression levels of LC3II/I and Beclin 1, as well as increased transcriptional levels of LC3, Beclin-1, Atg7, and Atg12, in periodontal ligament stem cells isolated from patients with periodontitis compared with healthy individuals (An et al., 2016).

### ROS induces autophagy by activating the Atg12-Atg5 complex

The Atg12-Atg5 conjugate is a ubiquitin-like protein complex that is essential for autophagophore elongation in autophagy (Otomo et al., 2013). A number of studies have provided evidence of the fine-tuning of Atg12-Atg5 in relation to the intracellular redox state (Mai et al., 2012). Pei et al. reported that the levels of Atg12-Atg5 were upregulated in a preodontoblast cell line (mDPC6T cells) after treatment with LPS for 6 h and 12 h but were downregulated after treatment with LPS for 24 h (Pei et al., 2015).

These findings show that autophagy can be induced in response to ROS through two master regulators of autophagosome biogenesis (mTORC1 and Beclin-1) and the Atg5-Atg12 complex, which also plays important roles in autophagosome biogenesis (Figure 2).

Autophagy is also crucial in mitochondrial ROS generation and scavenging, which is predominantly achieved by the release and activation of Nrf2 (Komatsu et al., 2010). Emerging evidence has indicated that Nrf2 and its target genes are crucial for maintaining cellular redox homeostasis in the attenuation of oxidative stress-associated periodontal destruction (Tamaki et al., 2014; Kataoka et al., 2016; Sima et al., 2016).

Collectively, progress in the field of redox regulation in autophagy has provided increasing details of the crosstalk mechanisms between ROS and autophagy. However, there is still no direct evidence demonstrating that the activation/inactivation of autophagy is triggered by redox regulation signaling in periodontitis. Hence, the precise process in periodontal tissues still needs to be elucidated. Whether ROS is an upstream signal of autophagy in periodontitis also requires further investigation.

## Conclusions

The physiologic and pathologic roles of ROS in the initiation and development of periodontitis have been studied for decades (Battino et al., 1999; Patil et al., 2016). Accumulating evidence has demonstrated that although low levels of ROS can be beneficial, excessive generation of ROS and/or antioxidant deficiency results in tissue destruction in periodontal diseases (Di Meo et al., 2016). More importantly, studies have indicated that ROS function as upstream modulators of autophagy (Bhattacharya and Eissa, 2015). In turn, autophagy can regulate ROS through the Nrf2 signaling pathway (Komatsu et al., 2010). Furthermore, several lines of evidence suggest that autophagy is involved in the development of periodontitis (Tan et al., 2016). The relationship between ROS and autophagy has also been shown to be associated with processes of other diseases, such as cancer (He Z. J. et al., 2017). Based on the accumulated evidence, we speculate that redox regulation of autophagy may play an important role in the initiation and development of periodontitis. As a form of cytotoxic signaling, excessive generation of ROS can trigger aggravated inflammation, apoptosis, and dysregulated autophagy activity that induces periodontal dysfunction. Conversely, redox regulation of autophagy is an effective measure for antibacterial responses and is also associated with protecting periodontal cells from apoptosis. As there is insufficient evidence concerning the interplay between ROS and autophagy in periodontal dysfunction, it is very difficult to generalize the role of redox regulation in periodontitis-related autophagy. However, previous studies have suggested a dual role for the redox regulation of autophagy. These studies have demonstrated that ROS may play a crucial role in determining cell fate by inducing autophagy or apoptosis. Therefore, further studies are required to clarify the role and mechanism of redox regulation of autophagy in periodontitis, which may be particularly beneficial for developing new therapeutic strategies for periodontal disease.

## Author contributions

CL drafted the manuscript and prepared the figures. LM, YN, XL, and XZ drafted parts of the manuscript and prepared the tables. XX reviewed, edited, and approved the final version of the manuscript.

### Conflict of interest statement

The authors declare that the research was conducted in the absence of any commercial or financial relationships that could be construed as a potential conflict of interest.
